# Long-term effect of metformin on blood glucose control in non-obese patients with type 2 diabetes mellitus

**DOI:** 10.1186/1743-7075-7-83

**Published:** 2010-11-12

**Authors:** Hiroyuki Ito, Hidenori Ishida, Yuichiro Takeuchi, Shinichi Antoku, Mariko Abe, Mizuo Mifune, Michiko Togane

**Affiliations:** 1 Department of Diabetes, Metabolism and Kidney Disease, Edogawa Hospital, Tokyo 133-0052, Japan

## Abstract

**Background:**

We aimed to investigate the long-term effect of metformin on the blood glucose control in non-obese patients with type 2 diabetes mellitus.

**Methods:**

A retrospective study was performed in 213 patients with type 2 diabetes mellitus under the administration of metformin for more than one year. The clinical parameters were investigated for 3 years. The obese and non-obese individuals were defined as a body mass index (BMI) of 25 kg/m^2 ^or over (*n *= 105) and a BMI of less than 25 kg/m^2 ^(*n *= 108), respectively.

**Results:**

HbA1c levels were significantly decreased compared with those at the baseline time. The course of HbA1c was similar between the non-obese and the obese groups, while the dose of metformin required to control blood glucose was significantly lower in the non-obese group than in the obese group. The reductions in HbA1c were 1.2% and 1.1% at 12 months, 0.9% and 0.9% at 24 months, and 0.8% and 1.0% at 36 months in the non-obese and obese groups, respectively. BMI did not change during the observation periods. Approximately half of all patients required no additional antidiabetic agents or a reduction in other treatments after the initiation of metformin in either of the two groups.

**Conclusions:**

The present study demonstrated the long-term beneficial effect of metformin in non-obese (BMI < 25 kg/m^2^) diabetic patients. This effect appears to be maintained even after the observation period of this study, because metformin was limited to a relatively low dose in the non-obese group and the observed worsening in glycemic control over time can probably be attenuated by increasing the dose of metformin.

## Background

Metformin, one of the biguanide agents, has been recommended for the treatment of patients with type 2 diabetes mellitus according to the consensus algorithm published by the European Association for the Study of Diabetes (EASD) and American Diabetes Association (ADA), because it is economical, induces less weight gain and does not cause hypoglycemic attacks, in addition to its glucose-lowering effect [1]. The UK Prospective Diabetes Study (UKPDS) demonstrated that metformin is as effective as sulfonylurea to control the blood glucose levels of obese patients with type 2 diabetes mellitus. Metformin yielded a stable patient body weight, cardiovascular protection and a better patient survival rate compared with sulfonylurea or insulin therapy [2,3]. Therefore, metformin is now accepted as the first-line drug for the type 2 diabetic obese patients.

The body mass index (BMI) and body fat percent are different between Asians and Caucasians. The BMI is 3 - 4 units lower in the general population in Asian compared to Caucasians [4]. It is similar in Japanese diabetic patients, for example, the mean BMI was 22.9 kg/m^2 ^in men and 23.4 kg/m^2 ^in women in the Japan Diabetes Complications Study (JDCS), which was a nationwide multi-center prospective study of type 2 diabetic patients [5]. Therefore, the strategy for treating non-obese patients is considered to be important in the treatment of type 2 diabetes mellitus for Asians, including Japanese.

Although it has been reported that metformin is effective for non-obese diabetic individuals, the observation periods were relatively short, usually less than one year [6-12]. Because the effects of oral hypoglycemic agents (OHAs), including metformin, and insulin are gradually reduced as the treatment periods increase [2,13], the observation of metformin over a longer duration is necessary.

In the current study, we aimed to investigate the long-term effects of metformin on blood glucose control in non-obese Japanese patients with type 2 diabetes mellitus.

## Patients and Methods

**Figure 1:**
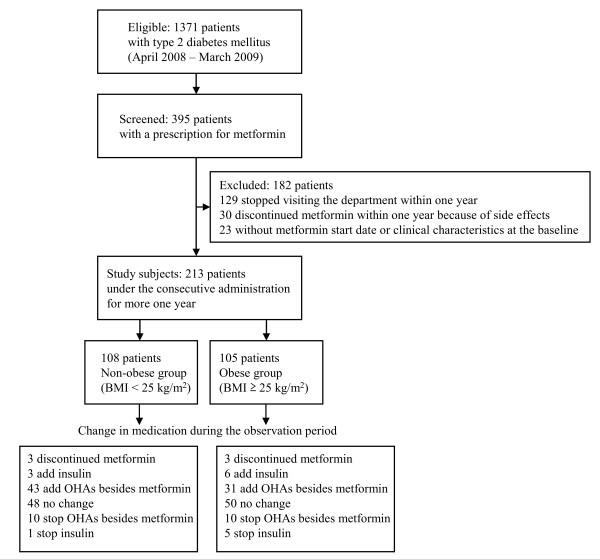
**Flow diagram of the population cohort**. BMI: body mass index. OHAs: oral hypoglycemic agents.

Three hundred ninety-five subjects who received metformin were conducted to be eligible for this study among 1371 patients with type 2 diabetes mellitus being treated in the Department of Diabetes, Metabolism and Kidney Diseases of Edogawa Hospital, Tokyo, Japan between April 2008 and March 2009 (Figure 1). Any individuals who had stopped visiting our department by changing the hospital or for other unspecified reasons for less than 1 year (*n *= 129) or who discontinued metformin within 1 year after the initiation because of side effects (*n *= 30), such as gastrointestinal symptoms and liver injury, were excluded from this study. The patients with an uncertain metformin treatment start date or without clinical characteristics at the baseline (before initiating metformin therapy) time were also excluded from the present series (*n *= 23). Finally, a retrospective study was performed in a population of 213 patients with type 2 diabetes mellitus under the consecutive administration of metformin for more than 1 year. The clinical parameters, including BMI, HbA1c level and the history of medication were investigated from the baseline (the initial date of metformin therapy) for 3 years.

All of the indications, doses and discontinuation of metformin were guided by each patient's physician. Although the optimization of the treatment, such as a dose increase of metformin, an addition/reduction of the other OHAs and an introduction of insulin therapy, was also judged by the chief physician, it was performed if the HbA1c levels remained higher than 7% or less than 5.8% for 2-3 months [14].

The obese and non-obese individuals were defined as having a BMI ≥ 25 kg/m^2 ^and a BMI < 25 kg/m^2^, respectively. Hypertension was defined as a systolic blood pressure ≥ 140 mmHg and/or a diastolic blood pressure ≥ 90 mmHg. The participants currently using antihypertensive medications were also classified as positive for hypertension. Serum total cholesterol, LDL-cholesterol, and HDL-cholesterol concentrations were measured with TBA-200 FR NEO using Determiner L TC II, Determiner L LDL-C, and Determiner L HDL-C instruments (Kyowa Medex Co., Ltd., Tokyo, Japan). Hyperlipidaemia was defined by serum concentrations of total cholesterol levels ≥ 5.7 mmol/L, LDL-cholesterol levels ≥ 3.6 mmol/L, or as patients who were already undergoing treatment with lipid-lowering agents. The triglyceride concentrations were not investigated in this study because fasting blood samples could not always be obtained for measurements. HbA1c levels were determined with a high performance liquid chromatography method using an automated HLC-723G7 analyzer (Tosoh Corporation, Tokyo, Japan) and calibrated by the Japan Diabetes Society (JDS) standard calibrators. The eGFR was calculated using the formula reported by Matsuo *et al *[15]. This equation originated from the MDRD study group [16] arranged for Japanese individuals, and it is recommended by the Japanese Society of Nephrology: eGFR (mL/min/1.73 m^2^) = 194  ×  Scr^-1.094^  ×  Age^-0.287^  ×  0.739 (if female).

### Statistical analysis

An analysis of variance (ANOVA) and the χ^2 ^test were used for between-group comparisons of the continuous and categorical variables, respectively. A paired *t*-test was conducted to determine whether there were any differences in the dose of metformin, HbA1c levels or BMI during the observation period compared to the baseline values. Pearson's univariate regression was performed to determine whether there was any association of reduced HbA1c with other clinical parameters. The odds ratio (OR) and respective 95% confidence interval (95% CI) were determined to examine the strength of the relationship between the requirement of additional glucose-lowering agents (OHAs or insulin) and the clinical parameters based on a multiple logistic regression analysis. Differences with *P*-values of less than 0.05 (two-tailed) were considered to be statistically significant. The statistical software package JMP, version 8.0 (SAS Institute, Cary, NC, USA), was used to perform all of the analyses.

## Results

Table 1 shows the baseline characteristics of the study patients. The age and the duration of diabetes mellitus were significantly higher and longer in the non-obese group than in the obese group. There was a greater frequency of patients without any antidiabetic agents prior to metformin administration in the obese group (31%) than in the non-obese group (16%). The blood pressure levels, the prevalence of hypertension and serum LDL-cholesterol concentrations were significantly higher in the obese patients than in the non-obese patients. The HbA1c levels and the initial dose of metformin were not different between the two groups.

**Table 1:** Baseline characteristics of the patients

	Non-obese	Obese	
	(n = 108)	(n = 105)	*P*
Body mass index (kg/m^2^)	22.7 ± 1.7	28.4 ± 2.9	<0.01
Age (years)	64 ± 7	59 ± 10	<0.01
Men (%)	49	52	0.63
Duration of diabetes mellitus (years)	12 ± 9	8 ± 7	<0.01
Current plus past smoking (%)	62	67	0.59
Current drinkers (%)	58	60	0.79
Therapeutic method for diabetes mellitus			
Diet only/OHAs/Insulin (%)	16/67/18	31/48/21	<0.01
Systolic blood pressure (mmHg)	132 ± 16	137 ± 16	0.01
Diastolic blood pressure (mmHg)	78 ± 10	81 ± 11	0.02
Hypertension (%)	61	87	<0.01
Total cholesterol (mmol/L)	5.3 ± 1.1	5.3 ± 1.1	0.71
LDL cholesterol (mmol/L)	2.9 ± 0.7	3.1 ± 0.8	<0.01
HDL cholesterol (mmol/L)	1.6 ± 0.5	1.4 ± 0.3	0.09
Hyperlipidaemia (%)	81	84	0.52
Haemoglobin A1c (%)	8.1 ± 2.0	8.1 ±1.7	0.90
Uric acid (μmol/L)	282 ± 84	302 ± 79	0.10
Serum creatinine (μmol/L)	67 ± 17	67 ± 15	0.71
Estimated GFR (mL/min/1.73 m^2^)	72.6 ± 17.3	75.5 ± 17.7	0.25
Initial dose of metformin (mg/day)	556 ± 110	566 ± 116	0.49

OHAs: oral hypoglycemic agents, GFR: glomerular filtration rate

**Figure 2:**
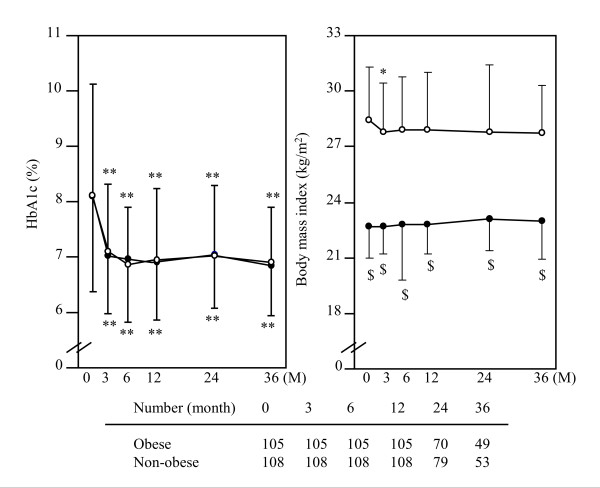
**Changes of HbA1c levels and body mass index in obese and non-obese patients with type 2 diabetes mellitus**. Data represent the mean ± SD. Open and closed circles represent the values in obese and non-obese individuals, respectively. * *P *< 0.05 and ** *P *< 0.01 vs. 0 M (initiation of metformin). ^$ ^*P *< 0.01 vs. obese.

**Figure 3:**
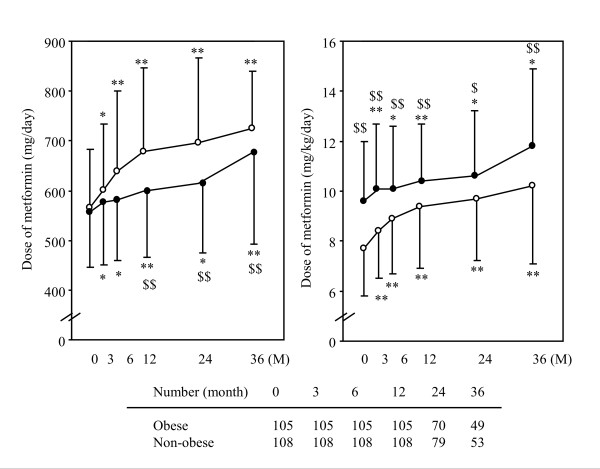
**Changes in the dose of metformin and those corrected by the patient's body weight**. Data represent the mean ± SD. Open and closed circles represent the values in obese and non-obese individuals, respectively. * *P *< 0.05 and ** *P *< 0.01 vs. 0 M (initiation of metformin). ^$ ^*P *< 0.05 and ^$$ ^*P *< 0.01 vs. obese.

During the observation period, HbA1c levels were significantly decreased compared with the baseline levels (Figure 2). The course of HbA1c was equal between the non-obese and the obese groups. HbA1c was reduced by 1.2% and 1.1% at 12 months, 0.9% and 0.9% at 24 months, 0.8% and 1.0% at 36 months in the non-obese and obese groups, respectively. Although the dose of metformin required to control the blood glucose levels gradually increased during the observation period (Figure 3), it was significantly lower in the non-obese group (598 ± 132 mg/day at 12 months, 614 ± 138 mg/day at 24 months and 677 ± 184 mg/day at 36 months) than in the obese group (678 ± 169 mg/day at 12 months, 696 ± 170 mg/day at 24 months and 724 ± 117 mg/day at 36 months). However, the dose of metformin after body weight correction was significantly higher in the non-obese group than in the obese group over the course of the observation periods (Figure 3). The values of BMI were not significantly different through all periods in the two groups (Figure 2).

The correlations of the clinical parameters to the reduction of HbA1c are shown in Table 2. Only HbA1c levels at the baseline time were significantly correlated with the reduction in each time of the observation period, although the duration of diabetes mellitus only showed a correlation at 1 year after the initiation of metformin.

**Table 2:** HbA1c reduction determinants by correlation coefficient

	1 year		2 years		3 years	
	
	*r*	*P*	*r*	*P*	*r*	*P*
Sex	0.06	0.42	-0.07	0.42	-0.15	0.14
Age	0.11	0.60	-0.02	0.79	0.14	0.16
Duration of diabetes	0.24	<0.01	0.16	0.10	0.15	0.21
Body mass index	-0.04	0.54	-0.04	0.61	-0.10	0.34
HbA1c at baseline	-0.81	<0.01	-0.73	<0.01	-0.82	<0.01
Hypertension	-0.09	0.19	-0.01	0.93	-0.01	0.91
Hyperlipidaemia	-0.02	0.75	-0.04	0.65	0.23	0.47
Estimated GFR	0.03	0.69	0.04	0.63	0.04	0.67
Therapeutic method	0.13	0.07	0.17	0.10	0.11	0.27
Initial dose of metformin	-0.11	0.11	0.01	0.94	-0.03	0.75
Metformin dose at 1 year	-0.07	0.32	0.08	0.36	-0.01	0.86

GFR: glomerular filtration rate.

Sex (man = 1, woman = 2), therapeutic method (diet = 1, oral hypoglycemic agents = 2, insulin = 3), hypertension (absent = 0, present = 1) and hyperlipidaemia (absent = 0, present = 1) were replaced with a number.

None of the patients were omitted from the study due to the side effects induced by metformin. Three patients discontinued metformin because of the progression of renal dysfunction in both obese and non-obese groups during the observation period (Figure 1). After starting the metformin treatment, three patients (3%) in the non-obese group and 6 patients (6%) in the obese group were introduced onto insulin therapy. Additional OHAs besides metformin were required in 43 (40%) of the non-obese and 31 (30%) of the obese patients. Forty-eight (44%) and 50 (48%) patients required no additional antidiabetic agents (other OHAs or insulin) or a reduction of the treatment in the non-obese and obese groups, respectively. The other OHAs besides metformin were either reduced or discontinued in 10 (9%) of non-obese and 10 (10%) of obese patients. One (1%) of the non-obese and 5 (5%) of the obese patients no longer required insulin therapy. The proportion of the changes in these treatment components was not significantly different between the non-obese and obese groups based on a χ^2 ^test. Table 3 shows the odds ratio for the requirement of additional agents (other OHAs or insulin) in the non-obese and obese patients with type 2 diabetes mellitus according to a logistic regression analysis adjusted by age, HbA1c at baseline and the therapeutic method (diet = 0 and oral hypoglycemic agents plus insulin = 1). The treatment using additional agents was significantly more frequent in the individuals with diet therapy alone at baseline than in those using other glucose-lowering drugs in both non-obese and obese patients. The use of additional agents was also required more frequently in the patients showing high HbA1c levels at the baseline among the obese group. The model adjusted by the duration of diabetes mellitus, HbA1c at baseline and the therapeutic method showed similar statistical results (date are not shown).

**Table 3:** Odds ratios for the requirement of additional agents in the non-obese and obese patients with type 2 diabetes mellitus determined by a logistic regression analysis.

	**Wald χ**^**2 **^**score**	OR (95% CI)	*P*
**Non-obese**			

Age	1.52	0.91 (0.91-1.02)	0.22
HbA1c at baseline	0.08	0.97 (0.77-1.19)	0.78
Therapeutic method	5.76	0.24 (0.07-0.74)	0.02

Obese			

Age	0.32	1.01 (0.97-1.06)	0.57
HbA1c at baseline	4.27	1.31 (1.02-1.71)	0.04
Therapeutic method	5.12	0.35 (0.14-0.87)	0.02

Adjusted by age, HbA1c at the baseline, and the therapeutic method (diet, oral hypoglycemic agents + insulin).

OR: odds ratio, CI: confidence interval.

**Figure 4:**
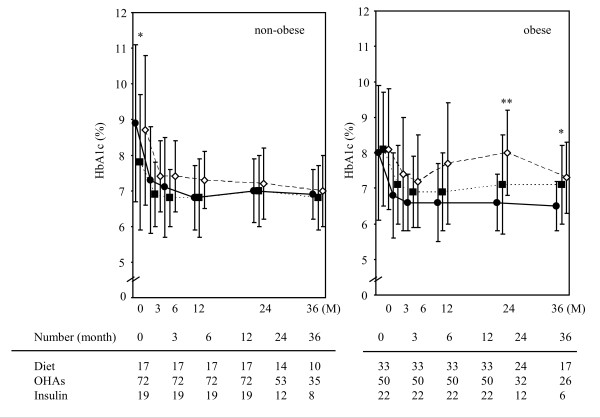
**Changes of HbA1c levels among the patient subgroups divided according to therapeutic methods prior to the initiation of metformin in the non-obese (left) and obese (right) patients with type 2 diabetes mellitus**. Data represent the mean ± SD. Closed circles, closed squares and open squares represent the diet, oral hypoglycemic agents (OHAs) and insulin therapies, respectively. * *P *< 0.05 and ** *P *< 0.01 among three treatment groups.

Figure 4 shows the changes of HbA1c levels among the subgroups as divided by therapeutic methods (diet only, OHAs and insulin) at the baseline in the non-obese and obese patients with type 2 diabetes mellitus. Although HbA1c was significantly lower in the subgroup using OHAs at the baseline time, the changes in HbA1c levels during the observation period were not significantly different among the three subgroups in the non-obese patients. On the other hand, HbA1c was significantly higher in the subgroups using OHAs or insulin at 24 and 36 months in the obese patients.

**Figure 5:**
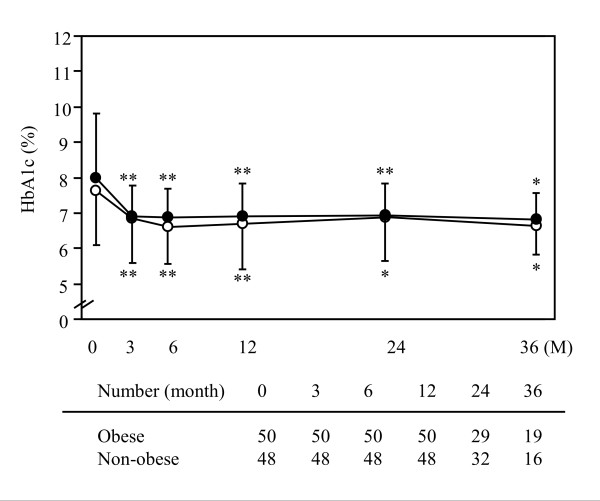
**Changes in HbA1c levels in patients who required no additional antidiabetic agents or a reduction in treatment**. Data represent the mean ± SD. Open and closed circles represent the values in obese and non-obese individuals, respectively. * *P *< 0.05 and ** *P *< 0.01 vs. 0 M (initiation of metformin).

To possibly minimize any interference caused by known changes in the other OHAs or insulin during the observation period, the changes in HbA1c levels were compared in the patients who required no additional antidiabetic agents or reduction in treatment between the non-obese (*n *= 48) and obese groups (*n *= 50). The course of HbA1c levels was not significantly different between the two groups (Figure 5). The reduction of HbA1c was 1.1% and 0.9% at 12 months, 1.1% and 0.8% at 24 months, 1.2% and 1.0% at 36 months in the non-obese and obese groups, respectively.

## Discussion

This study demonstrated the long-term beneficial effect of metformin therapy in non-obese (BMI < 25 kg/m^2^) patients with type 2 diabetes mellitus. Metformin induced a significant reduction in the HbA1c levels in non-obese patients. This effect was similar between non-obese and obese subjects who needed no additional antidiabetic agents or a reduction in other treatments during the observation period (Figure 5). Furthermore, similar glucose-lowering effects were obtained among the non-obese subgroups divided by the therapeutic methods prior to the initiation of metformin (Figure 4).

Although several studies have shown the advantage of metformin in non-obese diabetic patients [6-12], the observation periods were relatively short. Kaku *et al*. reported that the reduction of HbA1c by metformin was not different between 303 non-obese (0.9%, BMI < 25 kg/m^2^) and 300 obese (1.0%, BMI ≥ 25 kg/m^2^) Japanese patients with type 2 diabetes mellitus according to a prospective study for 12 months [6]. Hosokawa *et al*. retrospectively showed a significant 0.79% reduction in HbA1c levels in 58 patients with a BMI ≤ 22 kg/m^2^, 0.81% in 81 patients with 22 < BMI < 25 kg/m^2^, and 0.73% in 136 patients with 25 kg/m^2 ^≤ BMI 12 months after the initiation of metformin for type 2 diabetes mellitus [7]. Clarke and Campbell reported that metformin monotherapy (*n *= 98) was equally effective as chlorpropamide (*n *= 91), one of the sulfonylurea agents, on blood glucose control without HbA1c measurements, and that metformin was superior in the body weight control in non-obese patients with type 2 diabetes mellitus according to a prospective study for 1 year [8]. Yajima *et al*. demonstrated the metformin administration at a dose of 500-750 mg/day to be more effective in non-obese patients (*n *= 22, mean BMI was 25.6 kg/m^2^) with type 2 diabetes mellitus than in those treated at a dose of 150-300 mg/day of acarbose, an α-glucosidase inhibitors, in a crossover study conducted with 3-months treatment periods [9]. Lund *et al*. described that the glycemic regulation was equivalent between metformin and repaglinide, an insulin secretagogue, in a 4-month crossover trial in 96 non-obese (BMI ≤ 27 kg/m^2^) European patients with type 2 diabetes mellitus [10]. They also reported that the effect of metformin (*n *= 52) and repaglinide (*n *= 49) was not significantly different when combined with insulin for the treatment of non-obese patients, according to a randomized prospective study for 12 months [11]. Donnelly *et al. *showed the glucose-lowering effect of metformin to be very similar between the non-obese and obese patients with type 2 diabetes mellitus according to a study covering a period ranging from 3-12 months [12]. Ong *at al*. revealed that metformin was efficacious in non-obese subjects with type 2 diabetes mellitus (*n *= 136) according to a retrospective analysis of 16 years [17]. This is the first report to show the long-term effect of metformin among obese and non-obese patients with type 2 diabetes mellitus. Although the definition of non-obese was a BMI of less than 30 kg/m^2^, their investigation included 7% (*n *= 27) of individuals with a BMI < 25 kg/m^2 ^in their study subjects.

The glucose-lowering effect of metformin might be overestimated in the present study because the HbA1c levels were obtained from patients who had used metformin for more than one year. Individuals who stopped this treatment because of a lack of efficacy or side effects within one year were excluded. However, this limitation could be similar between the non-obese and obese groups.

The effects of OHAs and insulin gradually decrease as the treatment periods become longer [2,13]. Successive reduction of insulin from the pancreatic β-cells in patients with type 2 diabetes mellitus is considered to be a major factor for this attenuation of the drug effects [18-20]. The present study showed that a gradual increase of the metformin was required to control blood glucose levels during the observation period. However, the metformin dose increase was significantly lower in non-obese patients than in obese patients. Specifically, it may be possible to treat non-obese diabetic patients using a lower dose of metformin compared with obese patients. Donnelly *et al*. also reported that metformin was more effective in type 2 diabetic patients with a lower BMI [12]. However, the dose of metformin was higher in the non-obese patients than in the obese patients after the body weight correction in this study (Figure 3). It seems to be a paradox that a lower total dose of metformin while at the same time a higher dose corrected for body weight was found in the non-obese patients. In the present study, the duration of diabetes mellitus was significantly longer in the non-obese patients than in the obese patients. Furthermore, other glucose-lowering therapies, including OHAs and insulin were more frequently administered to the non-obese than to the obese patients. This underscored a potentially more severe stage of disease in the non-obese rather than in the obese patients. It is therefore possible that the endogenous insulin secretion was more strongly impaired in the non-obese than in the obese group, while the insulin resistance caused by, for example, visceral fat also was less in the non-obese than in the obese subjects of the present study. Although the parameters indicating insulin resistance and endogenous insulin secretion, such as serum or urine C-peptide concentration, were not evaluated in this study, the lower insulin resistance and secretion in the non-obese patients might have caused an increase in the requirement per body weight of metformin.

The levels of HbA1c were maintained within less than 7% during the 3-year observation period of this study. Additional OHAs or insulin treatment was necessary in 46 (43%) non-obese and 37 (35%) obese patients. It may also be caused by the attenuation of endogenous insulin secretion [18-20]. However, approximately half of patients required no addition or reduction of other treatments after the initiation of metformin in either group. Although the present study was not performed using metformin monotherapy, our results clinically demonstrated the advantage of metformin either in combination with the other antidiabetic agents or as a single therapy without any change in the treatment.

Metformin appears to maintain the glucose-lowering effect even after the observation period of this study had concluded, because the dose in the non-obese group was limited to 677 mg at 36 months of this study. The worsening in glycemic control over time observed in many patients with type 2 diabetes mellitus can probably be attenuated by increasing the dose of metformin because metformin reduces the HbA1c levels in a dose-related manner [21]. Metformin is a tolerable drug for patients if the dosage of the first year is completed, because no patients were omitted from this study due to side effects caused by metformin.

The results of this study have several limitations that must be considered. First, the findings are inherently limited by an inability to eliminate the effects caused by the other glucose-lowering agents, because our investigation included patients receiving a combination therapy of metformin and other antidiabetic drugs. It is difficult to study the long-term course of metformin monotherapy because of the attenuation of the effect as time passes [2,13]. Only 11 (3 non-obese and 8 obese) patients were treated with metformin monotherapy during the entire period, thus making it impossible to determine the significance of the therapy in these patients. To possibly minimize the interference caused by known changes in the other OHAs or insulin, the changes in HbA1c levels were compared in the patients who required no additional antidiabetic agents or reduction in treatment between the non-obese and obese groups (Figure 5). Although the change in the insulin dose is considered to be important among the patients receiving insulin treatment at baseline, this factor was not investigated in the present study. We therefore cannot exclude the possibility that some differences in the need for increasing the insulin doses between the non-obese and obese group of patients confounded our data with respect to the efficacy of metformin treatment during this study. This factor could also be a confounder even in those patients with no known (other) changes in the treatment. Second, this investigation is a retrospective study without the obvious criteria for the changes in treatment methods. Because the optimization of the metformin dose and the coordination of the treatment were dependent on each patient's physician, we considered that errors on the effects of metformin could have occurred. Third, the present study did not include a control group with a different treatment regimen, such as insulin secretagogues. Therefore, it is unknown whether the observed similarities and differences between the non-obese and obese patients were specific for these groups and/or specific effects of metformin therapy. Fourth, the associations of metformin with diabetic vascular events were not examined in this study. Furthermore, the risk factors for cardiovascular diseases such as blood pressure and serum lipid profile were not analyzed during the observation period because various antihypertensive agents and statins were used. The results of this study demonstrated the glucose-lowering effect of metformin in a group of non-obese patients with type 2 diabetes mellitus who continued such treatment for at least 1 year. Therefore, investigations to evaluate the effect of metformin therapy to potentially reduce the risk of developing diabetic complications also in non-obese patients with type 2 diabetes mellitus should be considered in the future.

## Declaration of competing interests

The authors declare that they have no competing interests.

## Authors' contributions

HIt and HIs contributed to the design of the study, analysis and interpretation of data, and drafting of the manuscript. YT, SA, MA, MM and MT contributed to interpretation of the data and critical revision of the manuscript. All authors have given their final approval of the submitted version of the manuscript.
